# Tree Species Diversity, Canopy Height, and Soil Nutrient Availability Shape Leaf Insect Herbivory in Zhoushan Archipelago, China

**DOI:** 10.1002/ece3.73582

**Published:** 2026-09-09

**Authors:** Yu Zhang, Enrong Yan

**Affiliations:** ^1^ Institute of Loess Plateau Shanxi University Taiyuan China; ^2^ Shanxi Technology and Business University Taiyuan China; ^3^ School of Ecological and Environmental Sciences East China Normal University Shanghai China

**Keywords:** ecosystem herbivory, insect feeding guilds, plant species diversity, soil nutrient effects, temporal variability

## Abstract

Plant‐insect interactions regulate ecosystem processes and productivity. Most studies have focused on the effect of plant diversity on insect herbivory in grasslands, croplands, and natural forests. However, the direction of this effect remains controversial. Unlike these ecosystems, island ecosystems possess distinct geographical features and provide natural experimental settings for studying trophic interactions. Nevertheless, the effects of plant diversity, island characteristics, and their interactions on insect herbivory in such environments have scarcely been explored. To test the relationships between plant diversity and total herbivory, as well as the herbivory of specific damage types, and to find the key determinants of insect herbivory in island ecosystems, 24 sample plots were established on 24 representative islands in 2019 in Zhoushan Archipelago, China. To ensure the reliability and repeatability of the results while minimizing inter‐annual variation in insect herbivory, 36 sample plots were set up on 30 different islands in 2021; all previous plots of 2019 were included in those of 2021. This study investigated insect herbivory in both years and measured plant species richness, climate factors, tree height, and soil nutrient availability in 2021. Polynomial (quadratic) regression was employed to examine the relationship between insect herbivory and plant diversity, and multiple linear regression (MLR), the stepwise regression model, and variation partitioning analysis (VPA) were used to identify significant patterns of insect herbivory. Our findings showed that tree species diversity was positively correlated with total herbivory and leaf mining in both 2019 and 2021, but only positively associated with sucking in 2019 and chewing in 2021. The extent of plant diversity effects varied among different damage types. In 2019, tree species diversity, tree height, soil nutrition, and their interactions collectively explained 60% of the variation in sucking. Tree species diversity, soil nutrition, and their interaction accounted for 32% of the variation in mining. But there were no significant relationships between climate factors and insect herbivory in this study. Synthesis. The results of this study partially support our initial hypothesis. Specifically, plant species diversity increased insect herbivory on islands, indicating that herbivorous insects on islands are almost generalists. However, the extent of effects among different damage types was inconsistent, which may stem from inter‐annual competition among herbivorous insect species. The discontinuous effects on sucking and chewing between 2019 and 2021 may also be ascribed to this. Additionally, in 2019, sucking was jointly regulated by plant species diversity, tree height, soil nutrition, and their interactions, with community structure and total nitrogen emerging as particularly important factors. For mining in the same year, plant diversity, soil nutrition, and their interaction were significantly correlated, indicating that soil environments with high nitrogen but low carbon and phosphorus favor mining species. However, climate factors showed no significant relationships with insect herbivory in both years, suggesting that microclimates may mediate external climate effects. These findings provide new insights into leaf insect herbivory patterns in island ecosystems.

## Graphical abstract

This study explores the relationship between plant diversity and insect herbivory in island ecosystems, specifically the Zhoushan Archipelago, China. The results show that tree species diversity positively correlates with total herbivory, as well as the herbivory of three damage types, and interactions between plant diversity, soil nutrition, and tree height influence different types of herbivory. However, climate factors did not significantly affect insect herbivory, supporting the Amplification Effect (AE) hypothesis in island ecosystems.

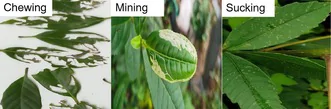

## 1 Introduction

Plant diversity is a key driver of ecosystem processes (Hooper et al. 2005; Hector et al. 2007; Byrnes et al. 2014). It has a significant bottom‐up effect on primary consumers (Duffy et al. 2007). Herbivorous insects are one of the most abundant among primary consumers, with plant leaves being their primary food source (Weisser and Siemann 2008). Plant identity and leaf functional traits can affect the visibility, palatability, and anti‐herbivore defenses of host plant leaves for herbivorous insects (Castagneyrol et al. 2019), thereby altering leaf insect herbivory. Furthermore, leaf performance plays a crucial role in shaping the damage types of herbivorous insects across evolutionary time (Muiruri et al. 2019). In this study, the herbivory damage types observed are chewing, sucking, and mining. Consequently, plant characteristics can lead to changes in leaf insect herbivory and insect damage types (Wright et al. 2004).

The patterns of leaf insect herbivory in different ecosystems vary substantially with plant diversity. Over the past decades, ecologists have put a lot of effort into exploring how plant diversity affects leaf insect herbivory (Castagneyrol et al. 2017). However, discrepancies among researchers remain unresolved. In croplands and grasslands, some ecologists argued that plant species diversity reduced chewing (Sobek et al. 2009). This negative relationship is known as the dilution effect (DE) (Shen et al. 2025). However, in tropical and subtropical forests, the literature reported that plant species richness amplified mining and chewing (Martini and Goodale 2020; Muiruri et al. 2019). This positive relationship is referred to as the amplification effect (AE) (Schuldt et al. 2010). Different from these ecosystems, island ecosystems exhibit distinct geographical features and provide a natural experimental setting for trophic interactions (Zhu 2020). However, there is limited research on insect herbivory conducted on islands.

Depending on the herbivory of a single damage type or multiple damage types, total herbivory, as an effective indicator, may reflect the effect of plant diversity (Andrew et al. 2012). The inconsistent directions and extent of plant diversity effects may be related to specialization (Jared and Anurag 2012). The amplification effect can result from the generalists benefiting from a greater variety of food resources. In contrast, the dilution effect can be attributed to changes in the likelihood of insect detection for the specialists. Diverse plant mixtures make it more difficult for the specialists to locate host plants (Boege and Marquis 2005; Barbosa et al. 2009). Total herbivory can compensate for the differences caused by the partial effects of the herbivory of different damage types. It would be an important indicator of insect herbivory. This study suggests that one reason for the inconsistency in previous plant‐insect findings is the lack of consideration of total herbivory and the herbivory of specific damage simultaneously.

Numerous studies have focused on the effect of insect herbivory on the density of young trees neighboring conspecific adults (Hyatt et al. 2003). However, some biotic and abiotic factors, including island characteristics, have rarely been taken into account as explanations for variation in insect herbivory (Castagneyrol et al. 2019). Building on previous island research, this study proposed perspectives concerning island characteristics: (1) The plant apparency postulate suggests that insect herbivory increases with tree height (Castagneyrol et al. 2013). For example, herbivory on downy birch ( *Betula pubescens* ) increased from small saplings to adult trees (Zverev et al. 2017). Other studies had also found that severe insect damage occurred in the lower strata of the canopy (Zhang et al. 2023). These differences may be related to the characteristics of the ecosystem. In island ecosystems, large trees can provide sheltered microhabitats for herbivorous insects, increasing their chances of survival. (2) The soil fertility postulate predicts that soil nutrient availability and insect herbivory interact with one another (Cuevas‐Reyes et al. 2004), although some studies have reported no significant relationships (Blue et al. 2011). Scarce soil nutrients in island ecosystems limit plant growth; conversely, fast‐growing plants are more vulnerable to leaf damage (Liu et al. 2024). (3) The climate postulate recognizes that climate has well‐documented effects on insect herbivory (Wright et al. 2017). Human‐induced climate change has been significantly altering biodiversity and ecological processes on islands (Tan et al. 2022). Climate factors are related to the reproductive processes and lifespan of insects (Wang et al. 2025). A study found that insect herbivory on birch leaves significantly increased with temperature but was not affected by rainfall (Poeydebat et al. 2021). In other findings, total herbivory was most effectively explained by variation in spring precipitation (Loughnan and Williams 2019). Moreover, the interactions among these drivers have been documented. The relative results indicated that the effect of plant diversity on insect herbivory was dependent on climatic conditions (Brezzi et al. 2017; Jactel et al. 2020). Insect herbivory can be altered by the interaction between plant diversity and tree height, and this interaction is indicative of the community structure (Zheng et al. 2019). However, the effects of plant diversity, island characteristics, and their interactions on insect herbivory have not been thoroughly explored in island ecosystems.

With clear boundaries, geographical isolation, island ecosystems are regarded as natural laboratories for ecological studies (Warren et al. 2015; Whittaker and Fernundez‐Palacios 2007). Numerous studies have been conducted on plants and animals on islands (Kreft et al. 2008; Hannus and Von 2008), but patterns of insect herbivory have hardly been explored. To better understand how plant species diversity, island characteristics and their interactions influence insect herbivory in island ecosystems, we formulated the following hypotheses for testing: Increased plant species diversity resulted in higher insect herbivory. However, the extent of these AEs among different damage types was inconsistent. In addition to plant species diversity, tree height, soil nutrient availability, climate factors and their interactions significantly influence total herbivory and the herbivory of specific damage types. This study took alternative hypotheses: Plant species diversity decreased total herbivory, as well as the herbivory of specific damage types. And the extent of these DEs was consistent. Additionally, insect herbivory was not significantly correlated with tree height, soil nutrient availability, climate factors or their interactions.

## 2 Materials and Methods

### 2.1 Study Site

**FIGURE 1:**
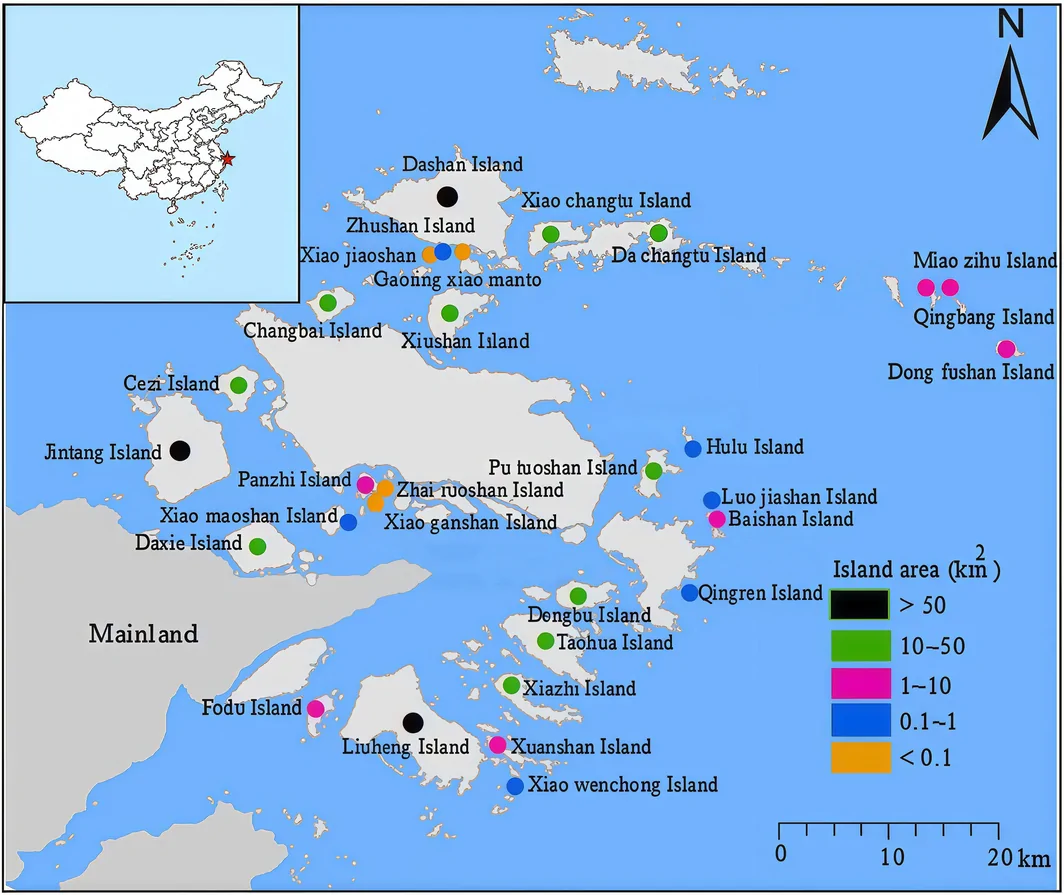
Geographical locations of the selected islands.

The study was conducted in the largest archipelago in eastern China, Zhoushan Archipelago (29°37.7′–30°50.9′ N, 121°25′–122°44.6′ E). This region has a subtropical monsoon climate, hot and humid in summer, dry and cold in winter. The mean annual temperature and precipitation are 17°C and 1600 mm, respectively. The mean annual wind speed ranges from 4.16 to 5.03 m/s (Zhu 2020). The main vegetation types are broad‐leaved forest, coniferous forest, shrubs, and grasses. The typical soils are mainly coastal saline soil, red soil, and coarse bone soil. Leaf‐insects are majorly chewers, suckers, and leaf‐miners. Damage from other types, such as rolling, has hardly been observed on leaves. After preliminary investigation, we selected 30 representative islands along the area (0.005–104.97 km^2^) and isolation (0.5–65 km) gradients. These selected islands feature typical island vegetation and leaf damage types in this area, as well as high biodiversity. The smallest island is Gaoting island, on the south side of Daishan island. The largest island is Daishan island (Figure 1).

### 2.2 Tree Species Sampling

From July to October, 24 sample plots were set up across 24 different islands in 2019. To verify the reliability and repeatability of the results and avoid the inter‐annual variation in herbivory, we established 36 sample plots across 30 different islands in the same period of 2021. All previous plots of 2019 were included in these of 2021. Considering higher diversity in large and mid‐size islands, we selected the sample plot covering most of the plant species on the island. We made two sample plots in Liuheng island, Panzhi island, Xiazhi island, and Xiushan island, and three sample plots in Daishan island, 2021 (Table S1). Due to differences in vegetation types (Chen 2017; Hu et al. 2016), plot size was 20 m × 20 m in forest, 10 m × 10 m in shrub. We also selected vegetation types that represent the overall vegetation landscape of the islands to sample. We used the statistical sample method of the British‐American schools in each sample plot, also known as checking every wood. We identified, labeled, and measured the woody plant individuals with a diameter at breast height ≥ 1 cm and higher than 0.5 m. Furthermore, we recorded the name of the sample plot, species name, diameter at breast height, base diameter, and tree height.

### 2.3 Determination of Soil Properties

This study adopted the soil protocol of Center for Tropical Forest Science (CTFS) (Condit 1998). Five samples (0–10 cm depth) were cored from each sample plot, and 180 soil samples were collected from 36 plots in 2021. We weighed the fresh soil sample, spread it evenly on a white paper, dried it in a ventilated place, and turned it over every 2 days. After the natural air‐drying completed, it was promptly ground and passed through a sieve (100 mesh). Then we put 0.15–0.20 g of soil in a tube (100 mL), added 5 mL of concentrated sulfuric acid and shook it evenly. A mixture of 1.5 g of sodium sulfate and copper sulfate was added, shook well, digested on a digester at 370°C, rinsed with a small amount of distilled water into a 5‐volume flask (500 mL). And we set the volume to 500 mL, filtered and clarified it before testing. At the same time, a blank control was performed. A fully automatic chemical analyzer (Smartchem 200, AMS Alliance, Italy) was inserted into every sample to determine the content of total phosphorus and total nitrogen of soil samples. In addition, 20 mg of the sieved sample was taken into a tin boat, weighed and tableted, and the total soil carbon was determined with a chemical elemental analyzer (Elementa, Germany).

### 2.4 Obtaining Climate Data

Based on the geographical coordinates of the sampling sites, climate data were extracted from the WorldClim database (https://www.worldclim.org/, version 2, resolution of 1 km). It provides monthly average data from 1970 to 2000. All extraction operations were conducted using the Extract Multi Values to Points tool in ArcGIS 10.1.

### 2.5 Herbivory Assessment

**FIGURE 2:**
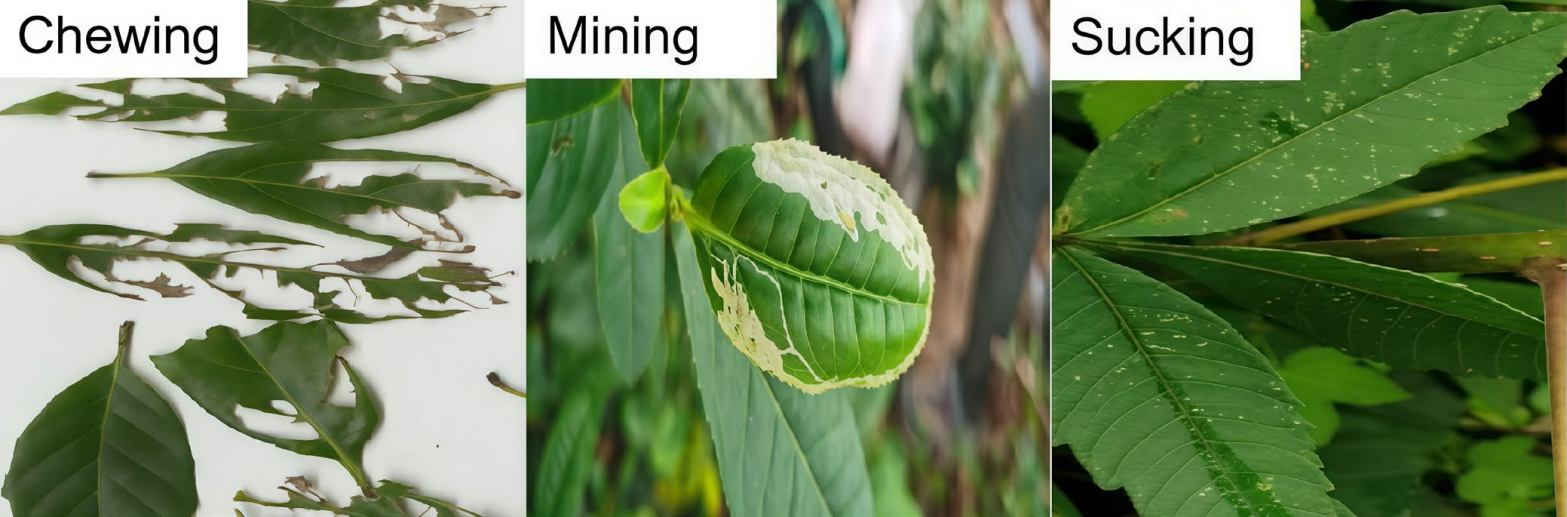
Characteristics of leaf insect herbivory on islands.

Considering the change of leaf damage throughout the year, herbivory assessment was performed once from July to October in 2019 and 2021. It results from the severest leaf damage occurring during this period (Muiruri et al. 2019). In each plot, at least three dominant plant species were selected, and common plant species were selected in high‐species plots to represent community‐level. We chose three to five individuals per species and randomly collected ten mature leaves from each individual. Then we promptly identified the damage type for each leaf (Figure 2). After identification, we put a transparent grid paper (the minimum grid unit is 1 cm^2^) upon the leaf to assess damage area; then the extent of damage was divided into 5 grades: (1) grade 0 (no herbivory): no insect herbivory trace on the leaf, (2) grade 1 (light herbivory): the area of herbivory traces accounting for 1%–25% of the entire leaf, (3) grade 2 (moderate herbivory): accounting for 26%–50% of the entire leaf, (4) grade 3 (severe herbivory): accounting for 51%–75%, (5) grade 4 (the severest herbivory): accounting for more than 75%. Finally, an estimate was obtained as the individual‐level insect herbivory expressed as a percentage (Muiruri et al. 2019). To avoid subjective bias and identify herbivory types concisely, leaf damage was assessed by one person only after one‐month training. As shown in Equation (1), the species‐level insect herbivory was defined as the average value of leaf damage per leaf within each species. It could eliminate differences between individuals.

(1) $$\text{Leaf damage in species}-\text{level}=\frac{\mathrm{sum}\;\text{of leaf damage}\;\mathrm{per}\;\text{leaf}}{\text{number of leaves}}$$

Community weighted mean (CWM) was used to estimate community‐level insect herbivory. Firstly, the diameter at breast height was converted to the cross‐sectional area of the breast, and the important value of each species was calculated, and the sum of the important value was guaranteed to be 100%. Secondly, the important values were matched to species‐level insect herbivory values. The CWM method can represent the functional trait values of dominant plant species within the community (Díaz and Lavorel 2007). In this study, traits were represented by leaf insect herbivory. Calculated according to Equation (2):

(2) $${\mathrm{CWM}}_{X}=\sum_{i=1}^{s}p_{i}t_{i}$$

where CWM*x* is the mean community weight of insect herbivory *x*, *s* is the number of species in the plot, *p*_*i*_ is the important value of species *i*, and *t*_*i*_ is the insect herbivory value of species *i*. Then, we added up these three individual‐level herbivory values to obtain individual‐level total herbivory, and repeat the above procedure to calculate the community‐level total herbivory values. The calculation is shown in Equation (3):

(3) Total Herbivory=Chewing+Sucking+Mining

### 2.6 Statistical Analysis

All analyzing processes were conducted in R language version 4.4.3 (R Core Team 2020). We used polynomial (quadratic) regression model to determine the effect of plant species richness on leaf insect herbivory in 2019 and 2021. Before analyzing the effect, we dealt the original data with statistical methods. To improve the normality of the data distribution, the data of leaf insect herbivory were standardized by the method of arc sine square root. Plant species richness was calculated by “rowSums” function (Table S2). Plant diversity, which varied among vegetation types, island size and plots on the sample island, influenced leaf insect herbivory within the same plot areas. In order to eliminate these effects, we used *rarecurve* in “VEGAN” package to estimate rarefied value of plant species richness (Oksanen et al. 2013).

Additionally, multiple linear regression (MLR) conducted by the “lm” function was used to reveal the effects of biological and abiotic factors on leaf insect herbivory. Biological factors included tree height and plant species richness; abiotic factors included mean annual temperature (MAT), mean annual precipitation (MAP), soil total nitrogen content (TN), total phosphorus content (TP), total carbon content (TC), carbon‐nitrogen ratio (C:N), carbon‐phosphorus ratio (C:P), and nitrogen‐phosphorus ratio (N:P). We used the stepwise regression model to select the significant determinants. The regression model was constructed as shown in Equation (4):

(4) $$\begin{array}{c} \text{Leaf damage}\sim \text{plant species richness}+\text{tree height}\\ +\mathrm{MAT}+\mathrm{MAP}+\mathrm{TC}+\mathrm{TN}+\mathrm{TP}+C:N+C:P+N:P \end{array}$$

And then, the equations with significant results were analyzed by variation partitioning analysis (VPA). Firstly, the significant variables were classified, and the relative contributions to leaf insect herbivory were calculated. VPA was implemented by the “VEGAN” package. The VPA model was shown in Equations (5 and 6):

(5) $$\begin{array}{c} {\text{Model}}_{\text{sucking}}\sim \text{varpart}\;\left(\text{Sucking},\sim \text{tree height},\right.\\ \sim \text{plant diversity}\sim \mathrm{TC}\left.+\mathrm{TN}+\mathrm{TP}\right) \end{array}$$

(6) $$\begin{array}{c} \;\text{Mode}l_{\text{mining}}\sim \text{varpart}\;\left(\text{Mining},\sim \text{plant diversity},\right.\\ \sim \mathrm{TN}+C:N\sim \mathrm{TC}+\left.\mathrm{TP}+C:P\right) \end{array}$$

## 3 Results

### 3.1 The Relationship Between Leaf Insect Herbivory and Plant Diversity

**FIGURE 3:**
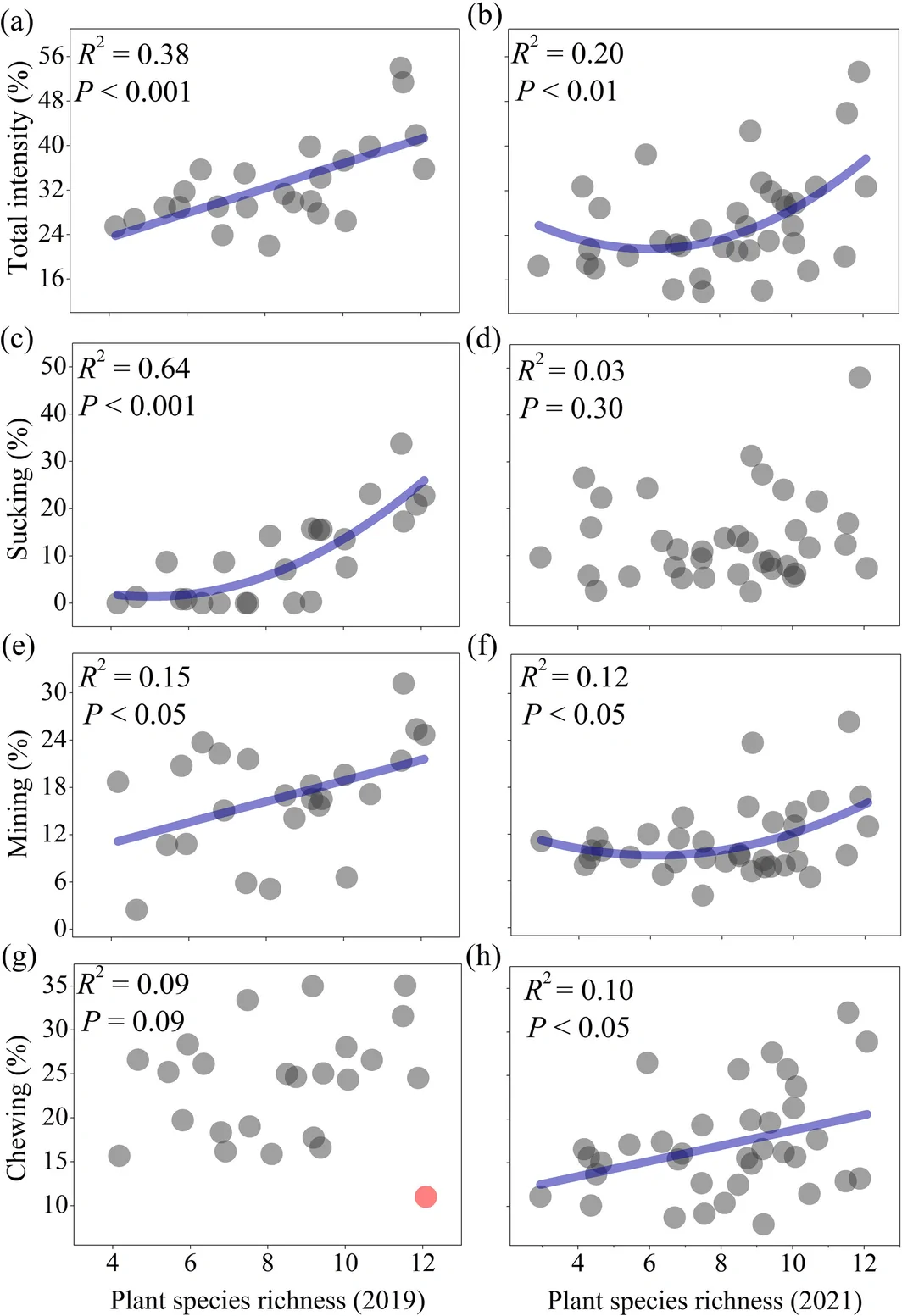
The relationships between plant species richness and intensity of different damage types and total herbivory in 2019 and 2021. The value of each point represents the community‐level herbivory, expressed as a percentage. The solid line indicates a significant relationship (*p* < 0.05). The red point is chewing in Oak community on Daxie Island, belonging to an abnormal point, so it is removed from calculation.

In 2019 and 2021, the effects of plant diversity on the intensity of different damage types and total herbivory were shown (Figure 3). In 2019, plant species richness increased the total herbivory (*R* ^2^ = 0.38, *p* < 0.001). As shown in Figure 3b, in 2021, plant species richness also increased total heirbvory (*y* = 0.004*x* ^2^–0.053*x* + 0.376, *R* ^2^ = 0.20, *p* < 0.01). From the perspective of single damage type, as shown in Figure 3c, in 2019, the sucking was the most sensitive to plant species richness (*y* = 0.005*x* ^2^–0.049*x* + 0.140, *R* ^2^ = 0.64, *p* < 0.001), as shown in Figure 3d, in 2021, there was no significant relationship between the sucking and plant species richness (*p* > 0.05). As shown in Figure 3e, in 2019, plant species richness increased the mining (*R* ^2^ = 0.15, *p* < 0.05), as shown in Figure 3f, in 2021, plant species richness also increased mining (*y* = 0.002*x* ^2^–0.023*x* + 0.165, *R* ^2^ = 0.12, *p* < 0.05). As shown in Figure 3g, in 2019, there was no significant relationship between chewing and plant species richness (*p* > 0.05), as shown in Figure 3h, however, in 2021, plant species richness increased chewing (*R* ^2^ = 0.10, *p* < 0.05).

### 3.2 The Relationships Between Biotic and Abiotic Factors and Leaf Insect Herbivory

The effects of biotic and abiotic factors on leaf insect herbivory were as shown in Table 1. In 2019, total herbivory was significantly related to biotic and abiotic factors (*R* ^2^ = 0.34, *p* < 0.05), among them, only plant diversity had a significant positive relationship with total herbivory (*p* < 0.05). From the perspective of single damage type, sucking was positively related to plant diversity, tree height and TN on sucking (*R* ^2^ = 0.60, *p* < 0.05). TC had a negative relationship with sucking (*p* < 0.05). The relationship between mining and these factors was significant (*R* ^2^ = 0.32, *p* < 0.05). Plant diversity, TN and C:N had significant positive relationships with mining (*p* < 0.05). There were significant negative relationships between the mining and TC, TP, C:P (*p* < 0.05). However, chewing had no significant relationships with any factors (*p* > 0.05). MAT and MAP had no significant relationships with leaf insect herbivory (*p* > 0.05).

**TABLE 1:** The relationships between intensity of different damage types and biotic/abiotic factors in 2019.

Relationships	Estimate	SE	*t*	*p*	*R* ^2^
Total intensity					
~Richness	0.016	0.004	3.568	**< 0.01**	0.34
~TC	−0.011	0.007	−1.423	0.174	
~TN	0.126	0.085	1.470	0.161	
~C:N	0.060	0.030	1.973	0.066	
~C:P	−0.002	0.001	−1.685	0.111	
~N:P	0.026	0.017	1.565	0.137	
~MAT	−0.065	0.051	−1.267	0.223	
Sucking					
~Richness	0.005	0.002	2.530	**< 0.05**	0.60
~TC	−0.002	0.001	−2.890	**< 0.05**	
~TN	0.039	0.015	2.680	**< 0.05**	
~TP	−0.13	0.067	−2.000	0.06	
~Tree height	0.009	0.003	2.910	**< 0.05**	
Mining					
~Richness	0.005	0.002	2.933	**< 0.01**	0.32
~TC	−0.010	0.003	−2.887	**< 0.05**	
~TN	0.134	0.045	2.971	**< 0.01**	
~TP	−0.351	0.148	−2.364	**< 0.05**	
~C:N	0.028	0.009	3.019	**< 0.01**	
~C:P	−0.0004	0.0001	−2.76	**< 0.05**	
Chewing					
~C:N	0.004	0.003	1.647	0.114	—

*Note:* SE represents the standard error, TC represents soil total carbon content, TN represents soil total nitrogen content, TP represents soil total phosphorus content, C:N represents carbon‐nitrogen ratio, C:P represents carbon‐phosphorus ratio, N:P represents nitrogen‐phosphorus ratio, MAT represents mean annual temperature. Bold parts represent significant relationships.

As shown in Table 2, in 2021, the relationship between total intensity and biotic and abiotic factors was significant (*R* ^2^ = 0.18, *p* < 0.05), among which, only plant diversity had a positive effect on total herbivory (*p* < 0.05). From the perspective of single damage type, the relationship between sucking and biotic and abiotic factors was not significant (*P* > 0.05). The relationship between mining and biotic and abiotic factors was significant (*R* ^2^ = 0.18, *p* < 0.05), the relationship between chewing and biotic and abiotic factors was significant (*R* ^2^ = 0.13, *p* < 0.05), among them, only plant diversity had significant positive relationships with the intensity of two damage types (*p* < 0.05).

**TABLE 2:** The relationships between intensity of different damage types and biotic/abiotic factors in 2021.

Relationships	Estimate	SE	*t*	*p*	*R* ^2^
Total intensity					
~Richness	0.010	0.003	2.984	**< 0.01**	0.18
~TC	−0.001	0.001	−1.630	0.112	
Sucking					
~Richness	0.005	0.003	1.724	0.094	—
~TC	−0.001	0.001	−1.929	0.062	
Mining					
~Richness	0.002	0.001	2.402	**< 0.05**	0.15
~TC	−0.0003	0.000	−1.674	0.104	
Chewing					
~Richness	0.003	0.001	2.333	**0.026**	0.13
~MAT	−0.024	0.017	−1.413	0.167	

*Note:* TC represents soil total carbon content and MAT represents mean annual temperature. Bold parts represent significant relationships, *p* < 0.05 indicates statistical significance, *p* < 0.01 indicates high statistical significance.

Furthermore, the significant factors affecting sucking and mining were classified. As for sucking, they were divided into three categories: plant diversity, tree height and soil nutrition content (TC, TN, TP). As for mining, they were divided into plant diversity, TN and C:N (positive effect factors), TC, TP and C:P (negative effect factors).

**FIGURE 4:**
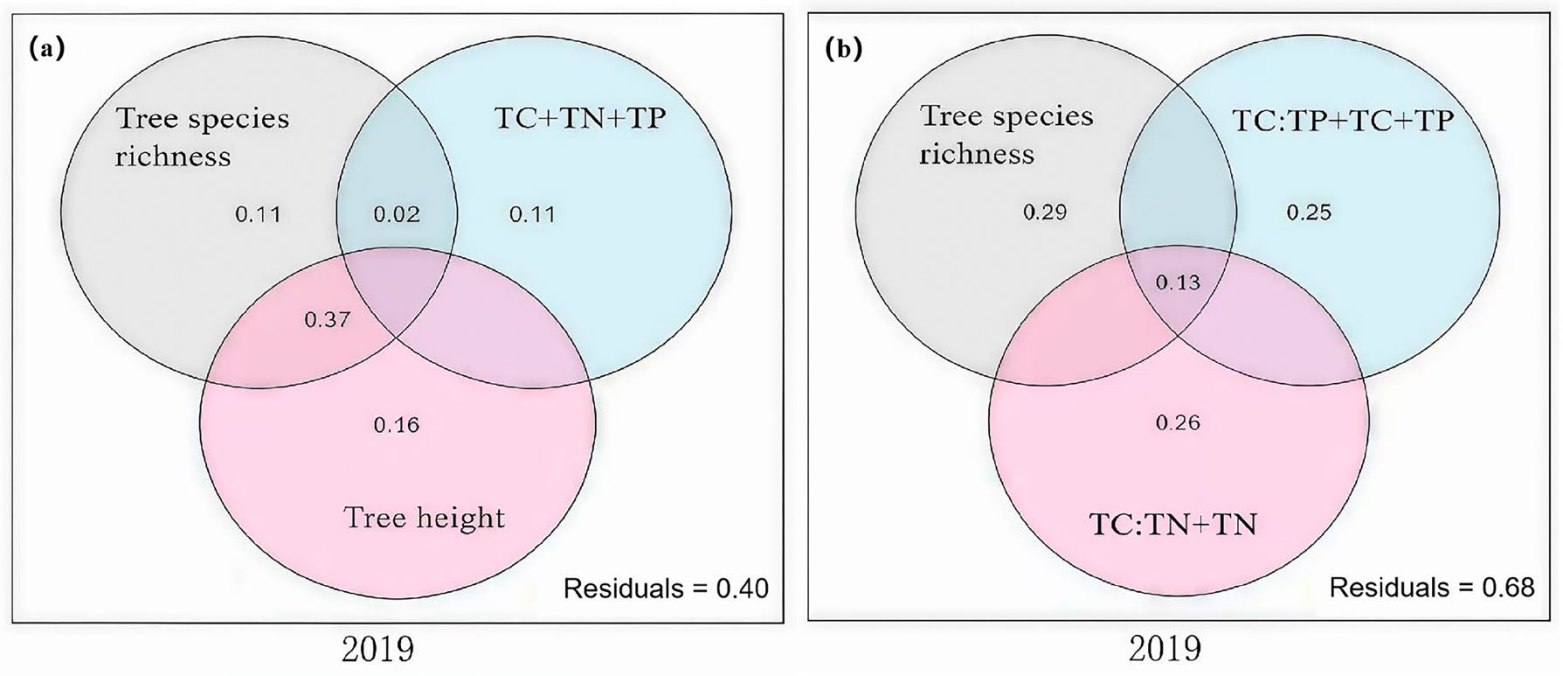
The variation partitioning analysis of sucking (a) and mining (b) by different factors in 2019. TC represents soil total carbon content, TN represents total nitrogen content, TP represents soil total phosphorus content. TC:TP represents carbon‐phosphorus ratio, TC:TN represents carbon‐nitrogen ratio.

The results of VPA (Figure 4a) revealed that 60% of the variation in sucking had been explained. The contributions of all categories were as follows: tree height accounted for 16%, plant diversity accounted for 11%, and soil nutrition content accounted for 11%. The interaction between plant diversity and tree height (37%) was ranked as the largest contributor; the interaction between plant diversity and soil nutrition content accounted for 2%. The interaction between tree height and soil nutrition content was not significant, and there was no significant interaction among the three categories.

The results of VPA (Figure 4b) showed that 32% of variation in mining had been explained. The contributions of all categories were as follows: plant diversity accounted for 29%, TN and C:N accounted for 26%, TC, TP, and C:P accounted for 25%. The contribution of the interaction among three categories was 13%.

## 4 Discussion

### 4.1 The Effects of Plant Diversity on Total Herbivory and the Herbivory of Three Damage Types

In 2019, the total herbivory ranged from 4.8% to 26.4%, positively related to plant diversity. This suggests that the dominant herbivorous insect species on Zhoushan islands might be polyphagous. Plant diversity explained 64% of the variation in sucking. Sucking was weaker than other types of herbivory, ranging from 0% to 11%, suggesting that sucking species are at a competitive disadvantage in acquiring food resources. This implies that plant diversity can be the key factor driving sucking (Vehviläinen et al. 2007).

Meanwhile, mining ranged from 0.1% to 9.4%, with only 15% of the variation explained by plant diversity, suggesting that other factors are likely more influential (Silva and Neves 2014). One possible explanation is that leaf‐miners preferentially feed on the tissue beneath the leaf epidermis, and the nutrient content within these tissues may account for a larger portion of the variation in mining (Gaston et al. 2004). It might also result from plant defensive mechanisms having a greater impact on mining damage than on herbivory of other damage types, as miners are entirely encapsulated within the plant.

This study also found that chewing contributed the most to total herbivory, ranging from 1.2% to 11.8%, indicating that chewing species are the dominant herbivorous group. However, chewing was not significantly affected by plant diversity, possibly due to other limiting factors, such as natural predators. Large‐bodied chewing species, such as *Lepidopteran* caterpillar larvae, may be more vulnerable to predation by birds and ants (Sam et al. 2023).

In 2021, the relationships between plant diversity and the herbivory of three damage types and total herbivory differed from those observed in 2019. Total herbivory, ranging from 1.9% to 25.8%, remained significantly related to plant diversity. However, the variation in total herbivory explained by plant diversity decreased by 18%, suggesting that the effect of plant diversity on insect herbivory had weakened over time. Mining ranged from 0.2% to 6.9% and was significantly correlated with plant diversity. However, the variation in mining explained by plant diversity decreased by 3%. Compared to 2019, sucking was slightly higher than mining but was not significantly related to plant diversity. Chewing, ranging from 0.6% to 10%, showed a positive relationship with plant diversity. Although chewing declined in 2021, it remained the highest component of total herbivory. Chewing herbivores are also more often generalists (highly polyphagous) compared to sucking and leaf mining herbivores. The reduction in their dominance may have resulted in chewing being regulated by plant species richness. The dominance of suckers increased more than that of leaf‐miners in 2021, indicating that suckers have advantages in exploiting food resources and are no longer limited by plant species richness.

In general, our results on the relationship between plant diversity and insect herbivory support our initial hypothesis that AEs occurred in this study. However, the extent of AEs was inconsistent, and the effects of plant diversity on sucking and chewing fluctuated across years.

### 4.2 The Effects of Biotic and Abiotic Factors on Total Herbivory and the Herbivory of Three Damage Types

A focus of research on leaf insect herbivory has been to clarify inducing factors (Andrew et al. 2012). This study took into account island characteristics, including climate, tree height, and soil nutrient availability. According to the results from 2019, sucking species are more sensitive to the interaction between tree height and plant diversity. This interaction can represent community structure (Zheng et al. 2019). It indicates that a complex community structure can increase sucking by protecting sucking species from the harsh island environment and from predators (Feller and Mathis 1997). However, a recent report found that tree height has a negative relationship with insect herbivory (Zhang et al. 2023). The effect of tree height may vary under specific environmental conditions. Sucking species on islands may face limitations in their ability to fly and reach and feed on the leaves of larger trees. Soil nutrition content can explain 11% of variation in sucking, especially total nitrogen, indicating that sucking species are nitrogen‐limited. High soil nitrogen content accelerates plant growth, leading to an increase in the abundance and richness of sucking species (Blue et al. 2011; Wu et al. 2023). Although the result for total soil carbon was significant, the correlation coefficient was weak. Therefore, this study cannot establish a definitive effect of total soil carbon on sucking.

According to the results from 2019, severe mining outbreaks are more likely to occur in high nitrogen and low carbon and phosphorus soil environments. Carbon‐to‐nitrogen ratio (C:N) and total nitrogen can promote host plant growth. High nitrogen content within leaves of fast‐growing plants is subsequently utilized in the synthesis of leaf‐miners' bodies and facilitates their reproductive processes (Ge et al. 2013). This explains why mining increases with carbon‐to‐nitrogen ratio (C:N) and total nitrogen (TN). Conversely, total carbon (TC) and total phosphorus (TP) can reduce net productivity and hinder plant growth (Wang et al. 2014), thereby inhibiting mining (Li et al. 2025).

This study found that climate factors have no significant relationships with insect herbivory. Unlike global‐scale experiments (Leckey et al. 2014; Liu et al. 2024), this study was conducted at a local scale. Microclimate can mediate the effects of external climate factors, providing a better explanation for the variation in insect herbivory.

Overall, our results on the effects of biotic and abiotic factors can partially support our initial hypothesis: (1) Tree height, soil nutrition, plant diversity and their interactions simultaneously contributed to variance in sucking. (2) Soil nutrition, plant diversity and their interaction were significantly correlated with mining. However, climate factors were not significantly related to insect herbivory.

## 5 Conclusion

The findings of this study showed that plant species diversity can amplify total herbivory, as well as the herbivory associated with three damage types. However, this study revealed the extent of AEs was inconsistent. And AEs on both sucking and chewing exhibited significant fluctuations between the years 2019 and 2021. There can be herbivorous insect competition differences between years may account for much of variation. The results concerning sucking were significantly correlated with plant species diversity, tree height, soil nutrition and their interactions in 2019. Community structure and total soil nitrogen played vital roles in shaping the patterns of sucking. Our analysis also suggests that soil environments with high nitrogen but low carbon and phosphorus are favorable for mining species. However, climate factors showed no significant relationships with insect herbivory. It could be related to the effects of microclimate. In future experiments, we recommend that additional factors be investigated, including the presence of natural enemies and competition among different damage types of insect species. Additionally, to facilitate long‐term monitoring, we propose installing weather monitoring instruments on several representative islands.

## Author Contributions

**Yu Zhang:** data curation (equal), formal analysis (equal), investigation (equal), methodology (equal), project administration (equal), writing – original draft (equal), writing – review and editing (equal). **Enrong Yan:** conceptualization (equal), funding acquisition (equal), resources (equal), supervision (equal), writing – review and editing (equal).

## Funding

This work was supported by the National Natural Science Foundation of China (NSFC) Key Program, [Grant Number: 32030068].

## Conflicts of Interest

The authors declare no conflicts of interest.

## Supporting information


**Table S1:** Basic information of the surveyed islands.
**Table S2:** Basic information of the sampled plots (SP, Species richness; MAT, Mean annual temperature; MAP, Mean annual precipitation).

## Data Availability

All the required data are uploaded as Supporting Information.
