# Supplementary material for: Tree Species Diversity, Canopy Height, and Soil Nutrient Availability Shape Leaf Insect Herbivory in Zhoushan Archipelago, China
Source: Ecol Evol. 2026 Sep 9;16(9):e73582. doi: 10.1002/ece3.73582 (PMC13558230; doi:10.1002/ece3.73582)
Supplement: Supplementary file 1 [file ECE3-16-e73582-s001.zip › Appendices.docx]

## Appendices

***Table S1.*** *Basic information of the surveyed islands*

| **Island name** | **Langitude(E)** | **Latitude(N)** | **Area(km^2^)** | **Isolation(km)** | **Maximum elevation(m)** | **Number of plots** | **Main vegetation types** |
| --- | --- | --- | --- | --- | --- | --- | --- |
| Baisha Island | 122°27.1′ | 29°56.7′ | 1.6 | 30.5 | 92 | 1 | Evergreen shrub |
| Cezi Island | 121°56.4′ | 30°06.8′ | 14.1 | 2.5 | 252.7 | 1 | Deciduous broad-leaved forest |
| Daxie Island | 121°57.7′ | 29°55.4′ | 30.84 | 0.5 | 334 | 1 | Deciduous broad-leaved forest |
| Dachangtu Island | 122°20.3′ | 30°15′ | 33.56 | 38.5 | 288.5 | 1 | Deciduous shrub |
| Dengbu Island | 122°18′ | 29°52′ | 14.5 | 13.8 | 174 | 1 | Deciduous broad-leaved forest |
| Dongfushan Island | 122°46′ | 30°08′ | 3 | 65.2 | 321.6 | 1 | Evergreen shrub and grass |
| Fodu Island | 122°01.2′ | 29°44.3′ | 7.3 | 7.8 | 150 | 1 | Evergreen and deciduous mixed forest |
| Gaoting Island | 122°10.8′ | 30°14.6′ | 0.005 | 37.3 | 12.5 | 1 | Evergreen broad-leaved forest |
| Hulu Island | 122°25.4′ | 30°02.4′ | 0.931 | 31.5 | 89 | 1 | Deciduous shrub |
| Jintang Island | 121°54.4′ | 30°01.9′ | 77.3 | 3.2 | 455.7 | 1 | Evergreen broad-leaved forest |
| Liuheng Island | 122°05.5′ | 29°45.4′ | 97.9 | 5.3 | 300.1 | 2 | Evergreen and deciduous mixed forest |
| Miaozihu Island | 122°40.6′ | 30°12.2′ | 2.6 | 61.5 | 142.5 | 1 | Evergreen shrub and grass |
| Panzhi Island | 122°04.4′ | 29°59.7′ | 3.8 | 8.3 | 159.7 | 2 | Evergreen and deciduous forest |
| Qingbang Island | 122°42.2′ | 30°12.21′ | 1.41 | 63.6 | 131.6 | 1 | Evergreen shrub and grass |
| Taohua Island | 122°17.2′ | 29°48.0′ | 40.4 | 9.2 | 544.7 | 1 | Evergreen broad-leaved forest and Evergreen grass |
| Xiazhi Island | 122°14.8′ | 29°46.5′ | 17.3 | 13.6 | 207.4 | 2 | Deciduous broad-leaved forest |
| Xiaoganshan Island | 122°05.6′ | 29°58.4′ | 0.047 | 8 | 31.3 | 1 | Evergreen broad-leaved forest |
| Xiaomaoshan Island | 122°03.3′ | 29°56.6′ | 0.537 | 5.7 | 126.9 | 1 | Evergreen and deciduous mixed forest |
| Xiaochangtu Island | 122°15.8′ | 30°15.8′ | 10.92 | 38.2 | 299.6 | 1 | Deciduous shrub and grass |
| Xiushan Island | 122°10′ | 30°9.9′ | 24.26 | 11.3 | 192.4 | 2 | Deciduous broad-leaved forest and coniferous forest |
| Xushan Island | 122°14.1′ | 29°41.7′ | 6.9 | 19.1 | 142.3 | 1 | Deciduous broad-leaved forest |
| Zhairuoshan Island | 122°05.1′ | 29°56.6′ | 2.4 | 4.5 | 215.4 | 1 | Evergreen and deciduous broad-leaved forest |
| Changbai Island | 122°02.7′ | 30°11.5′ | 11.1 | 17.3 | 249.1 | 1 | Deciduous shrub |
| Zhushan Island | 122°8.6′ | 30°14.2′ | 0.091 | 35.9 | 41.5 | 1 | Deciduous broad-leaved forest |
| Daishan Island | 122°11.7′ | 30°15.9′ | 104.97 | 37.8 | 256.5 | 3 | Evergreen broad-leaved forest |
| Luojiashan Island | 122°26.7′ | 29°58.2′ | 0.375 | 30.6 | 101.7 | 1 | Deciduous broad-leaved forest |
| Putuoshan Island | 122°23.2′ | 30°00.7′ | 12.3 | 25.3 | 288.2 | 1 | Deciduous broad-leaved forest |
| Qingren Island | 122°25.3′ | 29°52.3′ | 0.179 | 26.8 | 45.2 | 1 | Evergreen broad-leaved forest |
| Xiaojiaoshan Island | 122°9.4′ | 30°14.3′ | 0.16 | 36 | 57.3 | 1 | Deciduous broad-leaved forest |
| Xiaowenchong Island | 122°13.7′ | 29°39.0′ | 0.27 | 23.1 | 88.6 | 1 | Evergreen broad-leaved forest |

***Table S2.*** *Basic information of the sampled plots（SP-Species richness; MAT-Mean annual temperature; MAP-Mean annual precipitation）*

| **Plot name** | **SP** | **MAT** | **MAP** | **Soil TC** | **Soil TN** | **Soil TP** | **dominant species** |
| --- | --- | --- | --- | --- | --- | --- | --- |
| Plot 1 | 13 | 16.84999967 | 1227 | 37.42 | 2.86 | 0.105 | *Syzygium buxifolium, Loropetalum chinense, Eurya japonica* |
| Plot 2 | 15 | 16.17499967 | 1217 | 30.67 | 2.16 | 0.095 | *Quercus fabri,Loropetalum chinense, Eurya japonica* |
| Plot 3 | 13 | 16.43333275 | 1279 | 26.69 | 2.33 | 0.234 | *Quercus acutissima, Quercus fabri, Diospyros kaki* |
| Plot 4 | 5 | 16.49166642 | 1148 | 24.16 | 2.03 | 0.084 | *Syzygium buxifolium, Loropetalum chinense, Platycarya strobilacea* |
| Plot 5 | 10 | 16.58333283 | 1108 | 25.76 | 2.76 | 0.180 | *Camellia longicaudata, Celtis sinensis, Albizia kalkora* |
| Plot 6 | 15 | 16.64166625 | 1169 | 26.26 | 2.68 | 0.189 | *Ailanthus altissima, Quercus acutissima, Ficus erecta* |
| Plot 7 | 14 | 16.29999975 | 1170 | 29.38 | 2.66 | 0.192 | *Eurya japonica, Dalbergia hupeana, Albizia kalkora* |
| Plot 8 | 17 | 16.54999983 | 1246 | 20.93 | 1.99 | 0.178 | *Loropetalum chinense, Eurya japonica, Rhododendron simsii* |
| Plot 9 | 7 | 16.58333317 | 1200 | 28.24 | 2.65 | 0.216 | *Eurya emarginata, Eurya japonica, Ficus erecta* |
| Plot 10 | 16 | 16.46666617 | 1265 | 29.56 | 2.82 | 0.24 | *Symplocos paniculata, Camellia longicaudata, Cinnamomum japonicum* |
| Plot 11 | 11 | 16.49166592 | 1160 | 27.09 | 1.85 | 0.099 | *Eurya japonica, Liquidambar formosana, Cinnamomum camphora* |
| Plot 12 | 11 | 16.78333317 | 1211 | 20.62 | 2.05 | 0.1 | *Cinnamomum japonicum, Mallotus japonicus, Pittosporum tobira* |
| Plot 13 | 16 | 16.2166665 | 1248 | 28.8 | 1.96 | 0.073 | *Quercus fabri, Cyclobalanopsis glauca, Lithocarpus glaber* |
| Plot 14 | 17 | 16.39166617 | 1274 | 27.44 | 2.2 | 0.26 | *Quercus fabri, Cyclobalanopsis glauca, Lithocarpus glaber* |
| Plot 15 | 17 | 16.56666633 | 1268 | 26.52 | 2.32 | 0.132 | *Cyclobalanopsis glauca, Eurya japonica, Loropetalum chinense* |
| Plot 16 | 12 | 16.82499958 | 1266 | 33.52 | 3.48 | 0.08 | *Cinnamomum japonicum, Broussonetia papyrifera, Gardenia jasminoides* |
| Plot 17 | 6 | 16.26666633 | 1164 | 16.3 | 1.54 | 0.088 | *Eurya japonica, Mallotus japonicus, Pittosporum tobira* |
| Plot 18 | 15 | 16.75833325 | 1274 | 42.16 | 3.55 | 0.282 | *Quercus fabri, Mallotus japonicus, Platycarya strobilacea* |
| Plot 19 | 10 | 16.75833325 | 1277 | 36.51 | 3.55 | 0.443 | *Quercus fabri, Dalbergia hupeana, Schima superba* |
| Plot 20 | 22 | 16.433333 | 1225 | 30.52 | 1.3 | 0.08 | *Camellia longicaudata, Cyclobalanopsis glauca, Myrica rubra* |
| Plot 21 | 9 | 16.38333275 | 1161 | 10.48 | 1.05 | 0.084 | *Albizia julibrissin, Cyclobalanopsis glauca, Ficus erecta* |
| Plot 22 | 6 | 16.64999958 | 1226 | 23.06 | 2.12 | 0.205 | *Eurya japonica, Mallotus japonicus, Eurya emarginata* |
| Plot 23 | 18 | 15.89166642 | 1304 | 41.44 | 3.01 | 0.143 | *Loropetalum chinense, Cyclobalanopsis glauca, Syzygium buxifolium* |
| Plot 24 | 21 | 16.45833275 | 1267 | 44.6 | 3.07 | 0.166 | *Ilex chinensis, Rhododendron simsii, Camellia longicaudata* |
| Plot 25 | 13 | 16.39166617 | 1253 | 30.03 | 2.72 | 0.155 | *Platycarya strobilacea, Loropetalum chinense, Rhododendron simsii* |
| Plot 26 | 8 | 16.71666625 | 1273 | 30.57 | 2.24 | 0.144 | *Camellia longicaudata, Ficus erecta, Liquidambar formosana* |
| Plot 27 | 4 | 16.50833308 | 1150 | 21.16 | 2.34 | 0.41 | *Elaeagnus pungens, Broussonetia papyrifera, Cudrania tricuspidata* |
| Plot 28 | 12 | 16.20833292 | 1274 | 14.54 | 1.25 | 0.08 | *Rhododendron simsii, Eurya japonica, Symplocos setchuensis* |
| Plot 29 | 10 | 16.54166617 | 1280 | 27.88 | 2.51 | 0.173 | *Ficus erecta, Cinnamomum japonicum, Eurya emarginata* |
| Plot 30 | 11 | 16.54999958 | 1149 | 18.11 | 1.43 | 0.06 | *Loropetalum chinense, Eurya japonica, Ficus erecta* |
| Plot 31 | 9 | 16.16666633 | 1193 | 7.38 | 0.87 | 0.067 | *Quercus fabri, Loropetalum chinense, Ficus erecta* |
| Plot 32 | 12 | 16.29999958 | 1185 | 23.05 | 1.90 | 0.121 | *Eurya japonica, Loropetalum chinense, Quercus fabri* |
| Plot 33 | 10 | 16.64166608 | 1251 | 28.27 | 2.81 | 0.182 | *Rhododendron simsii, Loropetalum chinense, Elaeagnus pungens* |
| Plot 34 | 12 | 16.46666658 | 1281 | 50.05 | 2.76 | 0.21 | *Loropetalum chinense, Eurya japonica, Lithocarpus glaber* |
| Plot 35 | 8 | 16.38333283 | 1182 | 19.37 | 1.85 | 0.177 | *Mallotus japonicus, Elaeagnus pungens, Quercus fabri* |
| Plot 36 | 5 | 16.50833308 | 1150 | 20.38 | 2.14 | 0.136 | *Celtis sinensis, Pittosporum tobira, Ficus erecta* |
